# Uncovering microbial life-history strategies under disturbance: a trait-based computational analysis of anaerobic systems

**DOI:** 10.1038/s41522-026-01067-8

**Published:** 2026-07-10

**Authors:** Soheil A. Neshat, Ezequiel Santillan, Stefan Wuertz

**Affiliations:** 1 Singapore Centre for Environmental Life Sciences Engineering, Nanyang Technological University, Singapore, Singapore 637551, Singapore; 2 School of Civil and Environmental Engineering, Nanyang Technological University, Singapore, Singapore 639798, Singapore

**Keywords:** Computational biology and bioinformatics, Ecology, Ecology, Environmental sciences, Microbiology

## Abstract

Trait-based approaches are helpful in simplifying ecosystem complexity to explore disturbance-diversity-function relationships. These frameworks classify organisms based on their functional characteristics—traits that influence growth, survival and reproduction—providing a mechanistic basis to understand how communities respond to changes in their environment. The application of these approaches has been successful in ecology, but to date has only been tested in a few microbial ecosystems, namely, soil microbial communities and aerobic bioreactors treating wastewater. Here, we employed Grime’s competitor–stress-tolerant–ruderal framework in replicated mesophilic anaerobic bioreactors exposed to a disturbance (biomass removal) with varied frequencies at a constant number of disturbance events for 90 days. Bioreactors were inoculated with sludge from full-scale anaerobic digesters and fed with a mixture of primary and waste activated sludge. A genome-resolved metagenomics approach was utilised to assess the microbial communities in terms of composition and functional potential. We found that communities across the disturbance range were clustered into three groups, suggesting the adoption of a three-way life-history strategy. This study demonstrates, for the first time, the applicability of trait-based life-history strategies in anaerobic microbial systems under disturbance using genome-resolved techniques, providing a new perspective for understanding and managing microbial ecosystems under disturbance conditions.

## Introduction

Microbially mediated processes, driven by complex and dynamic microbial communities, are fundamental to modern wastewater treatment. The operational performance of these processes is directly linked to microbial community structure, which is often disrupted by environmental and operational disturbances^1^. While taxonomic profiling offers some insights into these communities, it lacks sufficient predictive power^2^. Trait-based frameworks, which link community structure to ecosystem functions, offer a promising alternative for understanding and managing these complex systems^2,3^.

Trait-based frameworks reduce ecological complexity by grouping organisms according to functional traits—attributes that influence fitness-related processes such as growth, reproduction and survival. These frameworks typically map organisms onto conceptual axes that represent ecological trade-offs. Two-dimensional models, such as r/K selection^4^, generalists vs. specialists^5,6^ and copiotrophy vs. oligotrophy^7,8^ have been widely used to study systems comprised of large organisms such as marine and land animals. However, such models often fail to capture the ecological nuances of microbial communities, which exhibit unique features such as dormancy, horizontal gene transfer and rapid adaptation^9^. To address such limitations, Ho et al.^9^ proposed the adoption of Grime’s competitor–stress-tolerant–ruderal (CSR) framework, a three-dimensional trait-based model originally developed for plant ecology. This framework defines life-history strategies along three axes representing key ecological pressures: competition, stress and disturbance. Its multidimensional structure offers a more flexible and ecologically meaningful way to characterise microbial strategies in dynamic environments, making it increasingly relevant for microbial ecology^2^. The application of such frameworks in microbial systems is often operationalized through Community Aggregated Traits (CATs), which summarise the distribution of traits across all community members. CATs offer a powerful means of linking metagenomic data to ecological strategy, enabling community-level positioning within trait spaces such as the CSR triangle^10,11^.

In the CSR model, organisms are classified into three primary life-history strategies: competitors (C), which maximise resource acquisition and control; stress-tolerants (S), which maintain metabolic activity under high-stress conditions; and ruderals (R), which exhibit rapid growth but low resource-use efficiency^12^. C-strategists tend to dominate productive, stable environments, S-strategists thrive in unproductive or harsh conditions and R-strategists are favoured in frequently disturbed environments. The balance among these strategies is shaped by three ecological forces: biomass destruction (disturbance), biomass restriction (stress) and resource competition. Meta-analyses have supported the applicability of the CSR framework in microbial ecology, particularly in soil ecosystem^9,10,13–16^.

Although promising, the CSR framework is still relatively new in the context of engineered microbial systems. To date, it has only been applied to activated sludge bioreactors treating synthetic wastewater under disturbance conditions^11,17^. Such studies established an important precedent, demonstrating that a three-way trait-based framework can effectively link microbial community structure to function using metagenomics in controlled microcosm experiments. These studies also introduced and tested the hypothesis that undisturbed, press-disturbed and intermediately disturbed systems would favour C, S and R strategies, respectively. However, several limitations remain— including the use of synthetic wastewater (excluding immigration effects), reliance on read-based metagenomics (which limits taxonomic resolution and functional linkage), small reactor volumes (20-25 mL), unequal numbers of disturbance events across experimental groups, and a labour-intensive analytical pipeline. While the CSR framework has shown promise in aerobic systems, its applicability to anaerobic digestion remains uncertain. The present study probes the relevance of the CSR framework in anaerobic systems using genome-resolved metagenomics, which, to our knowledge, has not been previously reported. Anaerobic communities are inherently more complex—comprising both bacteria and archaea and relying on tightly coordinated syntrophic interactions^18^—which may make them more sensitive to disturbances and perturbations harder to interpret. Testing of the CSR theory in this context is therefore essential when evaluating its broader ecological relevance and engineering utility. We have recently introduced a new computational tool, MicroEcoTools^19^, to apply the CSR framework to experimental disturbance datasets in a reproducible fashion.

The aim of this work was to explore the CSR life-history strategy framework in anaerobic digestion bioreactors (also known as anaerobic digesters) fed with waste activated sludge—thus incorporating immigration effects—and exposed to varied frequencies of disturbance, while maintaining an equal number of disturbance events across treatment groups. To this end, replicate laboratory-scale (mesocosm scale) anaerobic digesters were operated for a duration of 90 days. The digesters were subjected to varied frequencies of disturbance at a constant incidence number. Disturbance refers to controlled biomass removal events, in which anaerobic sludge are periodically withdrawn at defined frequencies to induce transient reductions in biomass concentration while maintaining a constant reactor volume and organic loading rate. The microbial community composition and functional traits were analysed using a genome-resolved metagenomics approach. In addition to assessing community composition and trait distributions, we evaluated community-level functions to identify potential trade-offs induced by disturbance.

## Results

### Genome-resolved metagenomic reconstruction and community overview

Genome-resolved metagenomic analysis recovered a total of 1665 metagenome-assembled genomes (MAGs) from four co-assemblies generated from 12 samples, following dereplication and quality filtering performed independently for each co-assembly. All retained MAGs met at least the MIMAG medium-quality criteria (completeness > 50% and contamination < 10%).

Coverage-based mapping of quality-controlled reads indicated that the reconstructed MAGs collectively accounted for more than 52% of the metagenomic reads, demonstrating that a substantial proportion of the microbial community was captured by the genome-resolved dataset. This level of recovery is consistent with expectations for complex anaerobic digestion communities.

At the phylum level, recovered MAGs spanned a broad taxonomic diversity characteristic of anaerobic digestion systems, including *Chloroflexota*, *Bacteroidota*, *Firmicutes* (multiple lineages), *Proteobacteria*, *Actinobacteriota*, *Synergistota*, *Desulfobacterota*, *Patescibacteria*, *Cloacimonadota* and *Hydrogenedentota*, as well as archaeal phyla such as *Methanobacteriota*, *Halobacteriota*, and *Thermoplasmatota*. Together, these groups encompass key fermentative, syntrophic and methanogenic guilds underpinning anaerobic digestion processes.

Detailed taxonomic assignments and coverage-based relative abundances of all recovered MAGs are provided in a publicly available dataset deposited on Zenodo (https://doi.org/10.5281/zenodo.19550057)^20^.

### Disturbance frequency modulated the community-level functions

Community-level functional traits were influenced by the disturbance frequency (Table 1). The highest biogas yield (0.44 ± 0.03 L/g VS _removed_/day), solids content (volatile: 31.23 ± 0.61 g/L; total: 46.20 ± 0.40 g/L), total alkalinity (4.39 ± 0.12 g CaCO_3_/L) and sulphate concentration (29.63 ± 24.94 mg/L; high variability across replicates) values were observed in undisturbed reactors. The highly disturbed reactors displayed the highest percentage of volatile matter removal (VMR; 54.55 ± 0.77%). These reactors showed the lowest solids content (38.33 ± 0.91 g/L), ammonia (1291.59 ± 21.03 mg/L) and total alkalinity (2.924 ± 0.09 g CaCO_3_/L) values.

**Table 1:** Summary of ecosystem traits and functions (process performance) and statistical comparison across digesters receiving different frequencies of disturbance after five disturbance events

Physicochemical property	Disturbance frequency^a^				*P*-value WA^b^	BH^c^-adjusted *P*-value
	Undisturbed	Low	Medium	High		
Ammonia concentration (mg/L)	2261.66 (93.45)	1207.76 (14.31)	1951.69 (104.91)	1291.59 (21.03)	0.0002	0.0015
Soluble COD^d^ (g O_2_/L)	1.78 (0.17)	1.51 (0.13)	1.79 (0.24)	2.26 (0.084)	0.0067	0.0130
Total COD^d^ (g O_2_/L)	51.15 (2.57)	50.82 (2.40)	46.02 (2.55)	42.57 (0.54)	0.0144	0.0243
Total alkalinity (mg CaCO_3_/L)	4338.00 (119.52)	3544.67 (198.32)	3544.00 (86.42)	2924.00 (85.71)	0.0004	0.0018
pH	7.57 (0.04)	7.61 (0.07)	7.48 (0.10)	7.39 (0.11)	0.1580	0.2133
Total solids (g/L)	46.2 (0.40)	42.6 (0.30)	41.97 (0.95)	38.33 (0.91)	0.0006	0.0018
Fixed solids (g/L)	14.97 (0.83)	13.67 (0.51)	13.23 (0.59)	11.70 (1.35)	0.1040	0.1478
Volatile solids (g/L)	31.23 (0.61)	28.93 (0.81)	28.73 (0.64)	26.63 (0.45)	0.0026	0.0053
Acetate concentration (mg/L)	79.75 (5.66)	54.27 (8.51)	84.05 (11.08)	80.82 (14.22)	0.0591	0.0939
Propionate concentration (mg/L)	3.28 (0.68)	3.00 (0.52)	2.57 (0.82)	3.36 (0.68)	0.6960	0.7830
Butyrate concentration (mg/L)	0.71 (0.03)	0.64 (0.07)	0.56 (0.15)	0.6 (0.06)	0.1667	0.2144
Valerate concentration (mg/L)	0.29 (0.04)	0.26 (0.08)	0.3 (0.08)	0.27 (0.08)	0.9197	0.9197
CO_2_^e^ (%)	27.22 (1.13)	26.38 (1.28)	27.23 (1.21)	25.97 (1.77)	0.7289	0.7872
CH_4_^e^ (%)	56.49 (0.62)	57.88 (1.96)	55.73 (1.45)	54.95 (3.6)	0.5949	0.6983
Chloride concentration (mg/L)	62.47 (0.4)	54.25 (0.9)	54.38 (1.71)	64.32 (0.03)	0.0006	0.0018
Nitrite concentration (mg/L)	0.52 (0.56)	0.48 (0.29)	0.23 (0.12)	1.5 (0.13)	0.0016	0.0040
Nitrate concentration (mg/L)	4.03 (0.74)	4.95 (2.39)	4.92 (0.33)	4.9 (1.69)	0.5209	0.6393
Phosphate concentration (mg/L)	1143.52 (30.56)	896.18 (18.24)	1040.45 (30.78)	1111.37 (16.43)	0.0004	0.0018
Sulphate concentration (mg/L)	29.63 (24.94)	4.02 (0.95)	17.70 (1.65)	10.23 (2.04)	0.0018	0.0041
CODs removal (%)^f^	79.61 (1.99)	86.4 (1.19)	79.49 (2.71)	72.76 (1.01)	0.0007	0.0018
CODt removal (%)^f^	49.02 (2.56)	54.72 (2.14)	54.14 (2.54)	59.39 (0.52)	0.0007	0.0018
VS removal (%)^f^	40.01 (1.17)	50.00 (1.4)	44.81 (1.23)	54.55 (0.77)	0.0002	0.0015
TS removal (%)^f^	30.60 (0.6)	43.68 (0.4)	36.96 (1.43)	48.41 (1.22)	0.0000	0.0009
Biogas Volume (L)	9.08 (0.6)	9.21 (0.25)	8.60 (0.96)	9.57 (2.21)	0.8102	0.8413
Biogas yield (L/g VS removed/day)	0.44 (0.03)	0.29 (0.01)	0.37 (0.03)	0.33 (0.08)	0.0138	0.0243
VS removal: VS content in digester (g VS removed/g VS in the digester)	0.67 (0.03)	1.20 (0.04)	0.81 (0.04)	1.00 (0.06)	0.0002	0.0015
Biogas volume: VS content in digester (L/g of VS in the digester)	0.29 (0.02)	0.35 (0.01)	0.30 (0.04)	0.33 (0.07)	0.0845	0.1268

Values represent measurements taken 10 days after the final disturbance event.

^a^Mean value (standard deviation).

^b^Welch-ANOVA test.

^c^Benjamini-Hochberg.

^d^Chemical oxygen demand.

^e^Content in the biogas measured by gas chromatography.

^f^Removal calculated based on the feed input.

The communities disturbed at intermediate frequencies of disturbance showed higher performance variability compared to undisturbed and highly disturbed. While solids content values in those reactors were comparable (28.93 ± 0.81 and 28.73 ± 0.64 g/L), biogas production yield (0.29 ± 0.01 and 0.37 ± 0.03 L/g VS _removed_/day), VMR (50.00 ± 1.4 and 44.81 ± 1.23%) and ammonia concentration (1207.76 ± 14.31 and 1951.69 ± 104.91 mg/L) fluctuated. Notably, VMR was significantly higher in reactors exposed to a low frequency of disturbance (50.00 ± 1.4%) compared to undisturbed reactors (40.01 ± 1.17%). Furthermore, ammonia (1207.76 ± 14.31 mg/L) and sulphate (4.02 ± 0.95 mg/L) concentrations were the lowest in reactors disturbed at a low frequency of disturbance. The differences in community-level functions across experimental groups were statistically significant (*P* < 0.05) except for volatile fatty acids concentration (acetate, butyrate, propionate and valerate), pH, biogas volume and biogas composition (Table S1). Despite significant differences between alkalinity values across the experimental groups, the pH values were similar (7.39 ± 0.11–7.61 ± 0.07). All the reactors actively produced biogas and degraded volatile matter.

### Three-way life-history strategies suggested by community structure and functional traits

**Fig. 1:**
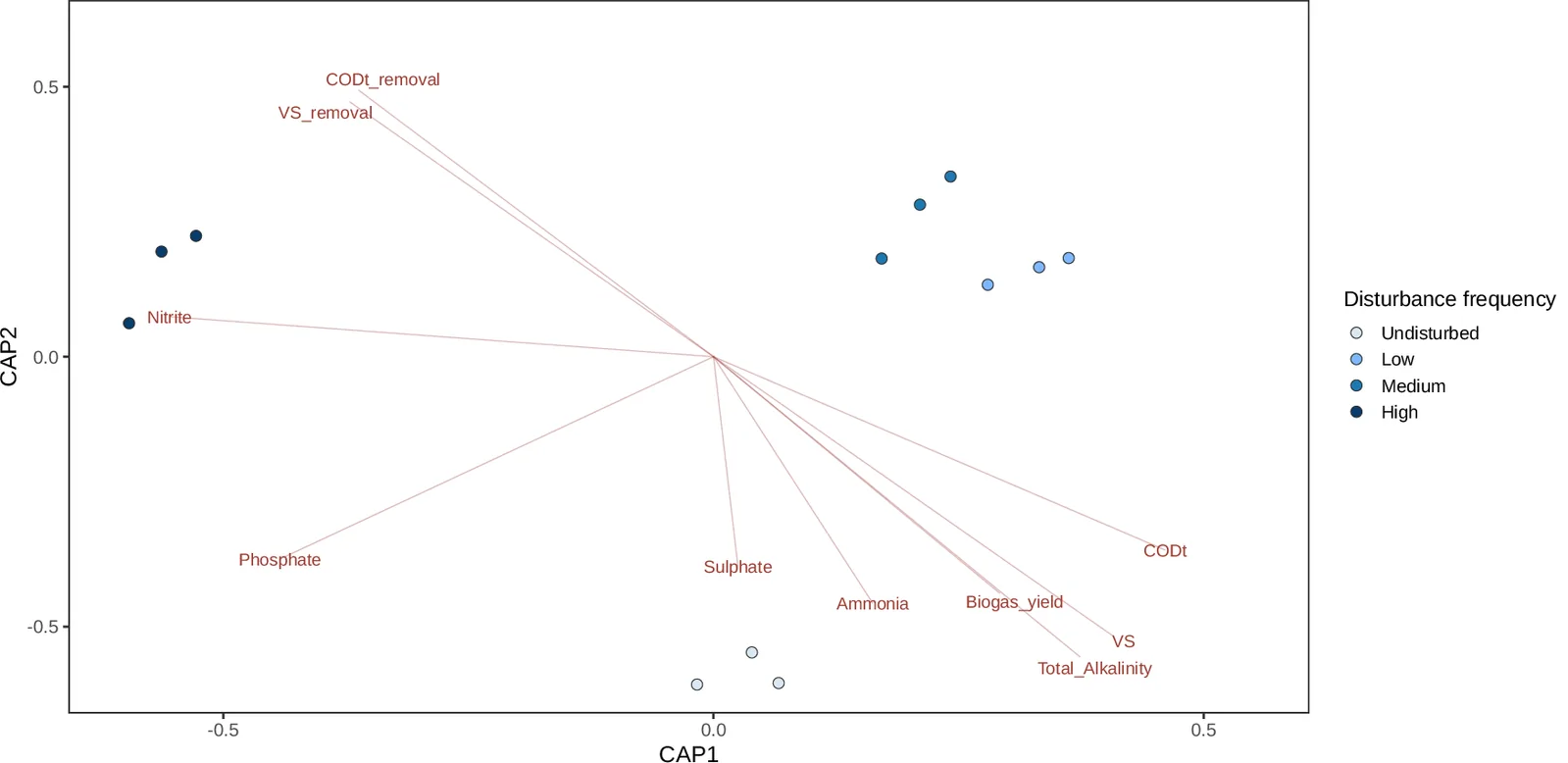
Canonical analysis of principal coordinates (CAP) summarising community-level differences in genome-resolved microbial composition across anaerobic digesters subjected to disturbance at varied frequencies. Each point represents a reactor community after five disturbance events, and colours denote disturbance frequency (undisturbed, low, medium and high). The biplot vectors show the significant community-level trade-offs (Welch-ANOVA *p* < 0.05) in terms of total COD removal (CODt_removal [%]), volatile solids removal (VS_removal [%]), total COD content of the digesters (CODT), volatile solids content of the digesters (VS), total alkalinity value, biogas yield calculated as the volume of biogas produced per gram of volatile solids removed, ammonia, sulphate and nitrite concentrations.

A canonical analysis of principal coordinates (CAP) (Fig. 1) on community structure highlights a three-way separation with undisturbed, intermediately disturbed and highly disturbed communities clustered. Communities exposed to intermediate disturbance levels form a distinct cluster, clearly separated from those under undisturbed or highly disturbed conditions. These three clusters create a triangular arrangement, highlighting the impact of disturbance frequency on community structure. Separation along the primary axis (CAP1) differentiates intermediately disturbed communities from highly disturbed ones, while the secondary axis (CAP2) sets undisturbed communities apart from the rest.

Permutational analysis of variance on the communities also reveals that the communities are significantly different from each other using disturbance frequency as explanatory variable (PERMANOVA-F = 14.044, *P* = 0.001). Multivariate homogeneity of groups dispersion test^21^ was insignificant, suggesting the difference highlighted by PERMANOVA was not because of heteroscedasticity (PERMDISP *F* = 0.1814, *P* = 0.9061). The difference in community structure coincides with different community-level traits (vectors shown on the CAP plot), suggesting adaption of different CSR strategies. The correlation analysis on the function-community structure dataset shows significant correlations between disturbance level, functions and community structure supporting the above arguments (Fig. S2).

Trade-offs were observed between solids content in the reactors (biomass production) and solids removal (consumption of organic materials for energy purposes). As highlighted by the correlation vectors, the disturbance frequency was positively correlated with the solids removal and negatively correlated with the solids content in the reactors. In addition, ion concentrations except for phosphate and nitrite were negatively correlated with the disturbance frequency.

### Effect of disturbance frequency on community structure at genus level

**Fig. 2:**
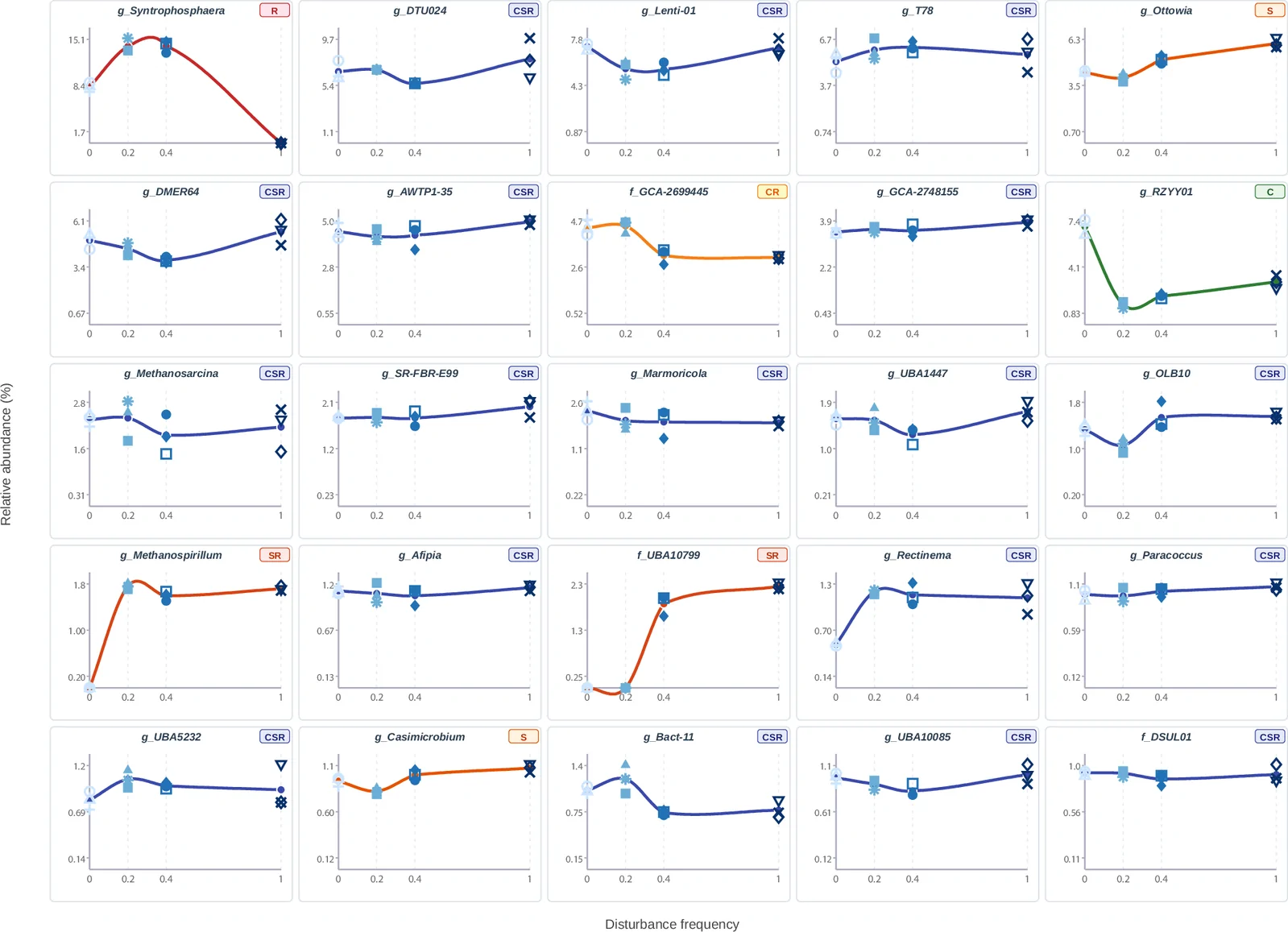
Relative abundance patterns of the 25 most abundant microbial taxa based on MAG coverage across replicate anaerobic digesters (*n* = 3) subjected to increasing disturbance frequency. Each panel represents a taxon classified at the highest confidently resolved GTDB taxonomic rank (genus or family). Points show individual reactor replicates within each disturbance treatment (0, 0.2, 0.4 and 1, corresponding to undisturbed, low, medium and high disturbance frequency, respectively). Symbols represent individual reactor replicates within each disturbance treatment, labelled as C (undisturbed; disturbance frequency = 0), L (low; 0.2), I (intermediate; 0.4) and H (high; 1). Trend lines illustrate abundance responses across the disturbance gradient and each taxon is annotated with its assigned CSR (competitor, stress-tolerant, ruderal, or intermediate) category. An interactive version of this figure, including full taxonomic annotation and all detected taxa, is available via the Zenodo repository^20^ (https://doi.org/10.5281/zenodo.19550057).

**Fig. 3:**
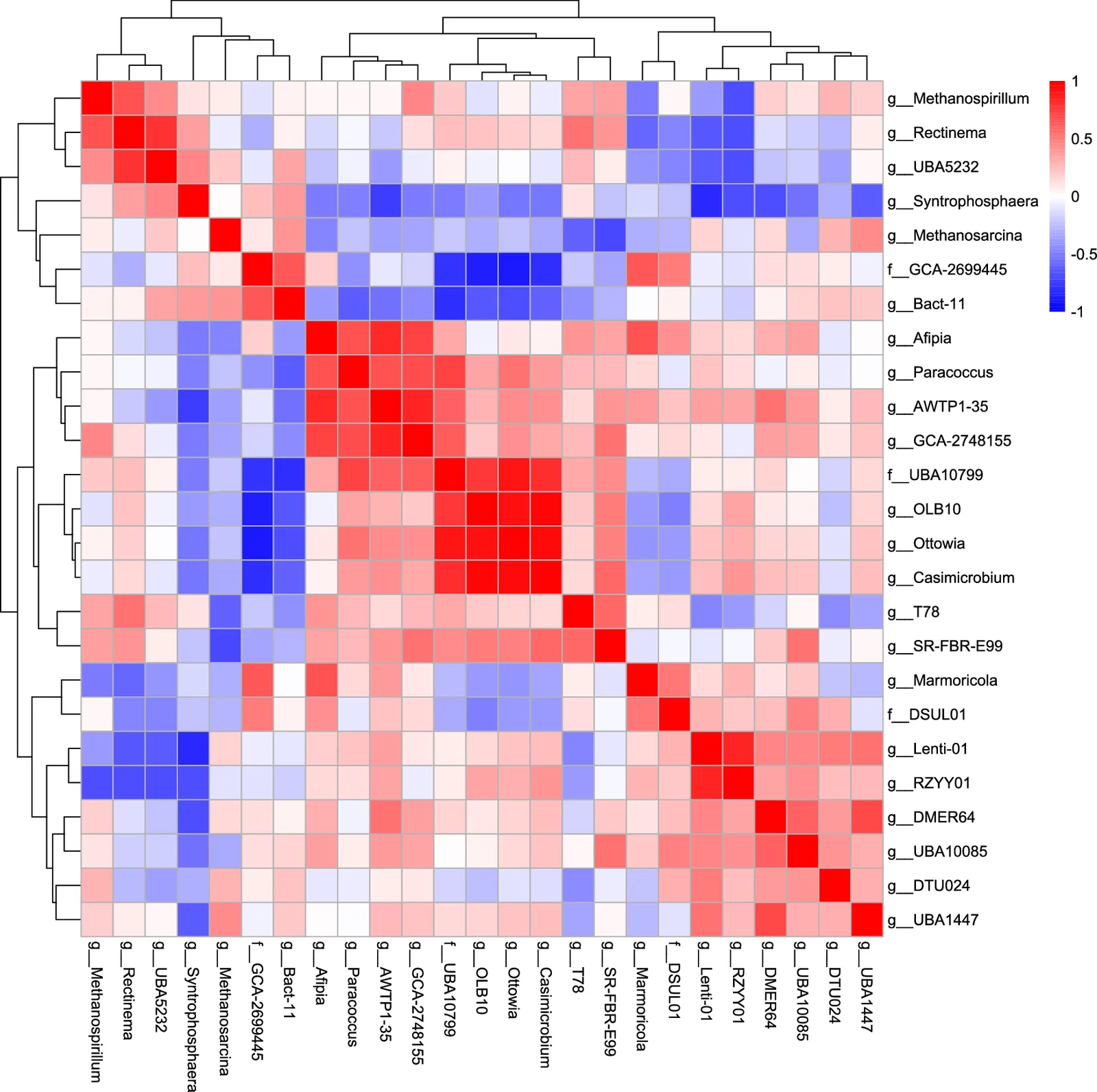
Spearman’s rank correlation heatmap for the 25 most abundant microbial taxa (MAGs), with double hierarchical clustering applied to both rows and columns. The colour of the tiles shows the Spearman’s correlation coefficient. Taxa represent genus- or family-level aggregates based on the highest confidently resolved GTDB assignment (g genus, f family).

The differences in community composition at the highest known taxonomy level across experimental groups is highlighted by scatter plots and heatmaps with double clustering (Fig. 2 and Fig. S3 for top 25, and Fig. S4 for top 100 taxa). The undisturbed conditions favoured taxa such as f__*Saprospiraceae*, g__*UBA8904* and g__*RZYY01*, while g__*Ottowia*, g__*MWFE01*, g__*Albidovulum*, f__*Rhodanobacteraceae*, g__*Sedimentibacter*, g__*Casimicrobium*, g__*OLB10*, g__*UBA1368* and g__*Sphaerochaeta* were more abundant in highly disturbed reactors. At low and medium disturbance frequencies, g__*Syntrophosphaera*, g__*Giesbergeria*, o__*UCB3* and g__*Phycicoccus-A* taxa were enriched. For the archaeal members of the community, g__*Methanosarcina* and *g__Methanothrix* were the dominant methanogens in all reactors (no difference across experimental groups), while g__*Methanospirillum* was only found in disturbed reactors. The differences in the relative abundance of g__*Syntrophosphaera*, g__*DTU024*, g__*Lenti-01*, g__*Ottowia*, g__*DMER64*, f__*GCA-2699445*, g__*RZYY01*, g__*OLB10*, g__*Methanospirillum*, f__*UBA10799*, g__*Rectinema* and g__*Casimicrobium* taxa across experimental groups were significant based on Welch-ANOVA via the CSR_assign function^19^ (Supplementary Table S2). In addition, there were strongly significant correlations between the community members, resulting in three large clusters (Fig. 3 and Fig. S4). In total, the majority of recovered MAGs could be assigned to one of the CSR or inter-class categories, with detailed counts and category distributions provided in Supplementary Table S2.

### Functional potentials enriched by varying frequency of disturbance

**Fig. 4:**
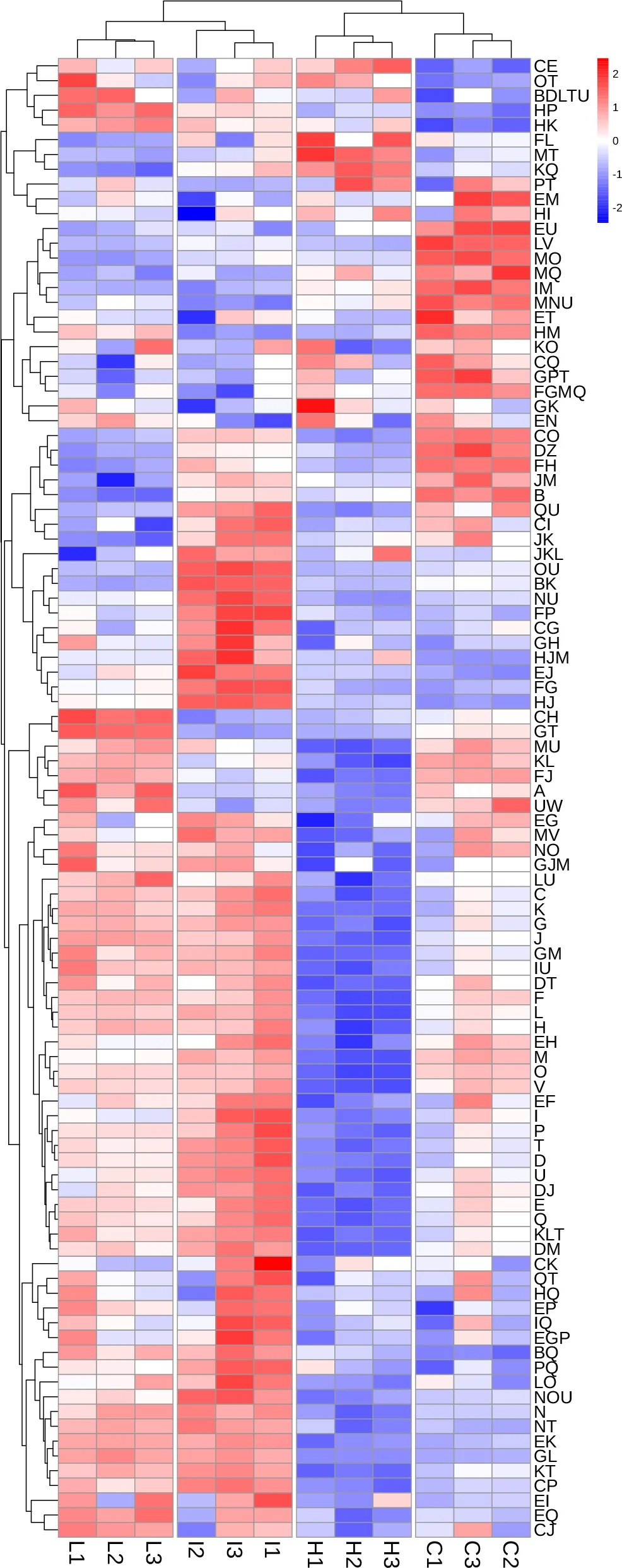
Abundance of the 100 most common COG categories derived from MAG-annotated genes and quantified using coverage-based mapping across experimental groups. Only the annotated genes with at least 10X coverage were included in this analysis. The x-axis shows the frequency of disturbance (C undisturbed, L low, M Medium, H High; numbers refer to the replicate digesters). The y-axis presents the COG categories (see Supplementary Table S5 for COG category descriptions). The tile colours represent weighted counts that were Z-scale normalised across reactors for each COG category—meaning each count had its category’s average subtracted and was then divided by that category’s standard deviation. Full COG category names and their broader functional classifications are provided in Supplementary Table S5.

Functional trait profiles diverged across disturbance regimes, with intermediately disturbed communities showing distinct relative patterns from undisturbed and highly disturbed groups. These profiles were analysed as relative, community-level indicators of functional allocation, rather than as organism-specific metabolic traits, to examine how disturbance frequency shapes functional patterning across anaerobic communities. Cluster analysis of COG category abundances revealed a clear separation, with intermediate communities forming a cohesive group distinct from the other two (Fig. 4, Fig. S5). This pattern was supported by PERMANOVA (*F* = 52.04, *P* = 0.001), indicating a significant effect of disturbance frequency on functional composition, while PERMDISP confirmed homogeneity of group dispersions (*F* = 0.54, *P* = 0.67; Tables S3, S4). Functional divergence was further reflected in the abundance of carbohydrate-active enzymes (CAZy), which also varied significantly among groups (Fig. S6; Tables S3, S4). To explore life-history strategy patterns, COG and CAZy categories were classified under the CSR framework using the MicroEcoTools R package (Tables S5, S6).

### Network analysis highlights a three-way life-history strategy in communities under varied frequencies of disturbance

**Fig. 5:**
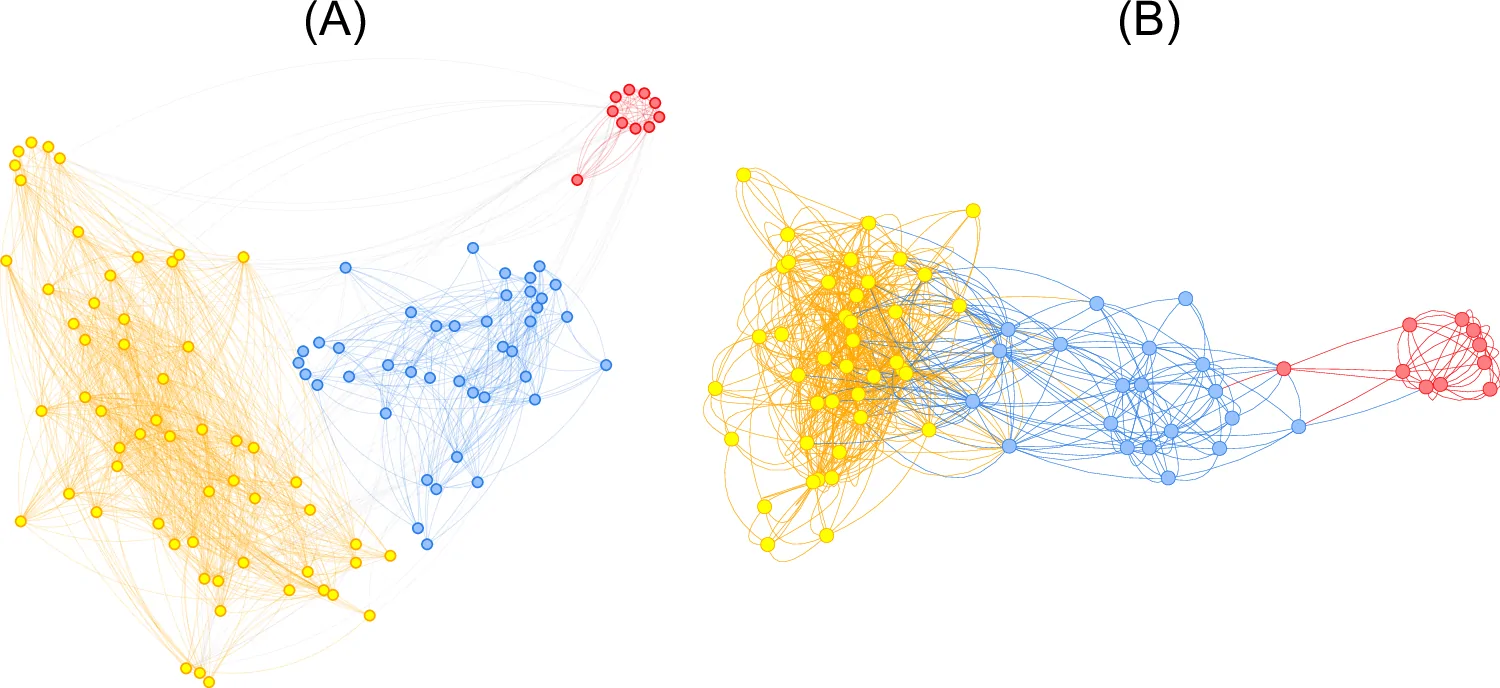
Correlation network analysis illustrating modular structure in microbial taxa and functional potentials inferred from genome-resolved metagenomic data. Networks were constructed using significant positive Spearman correlations (*p* < 0.05 and Spearman’s *ρ* > 0.5) based on MAGs coverage profiles. **A** Network of microbial taxa represented at the genus level. **B** Network of COG functional categories annotated in MAGs with at least 10X coverage. Nodes represent MAGs at the genus level (**A**) or COG functional categories (**B**) and edges indicate significant positive correlations. Node and edge colours denote network modules identified independently within each network using a modularity-based community detection algorithm via the resolution R package^56^. These colours highlight emergent co-variation structure within each network and do not represent predefined ecological classifications (e.g. CSR categories) or trophic guilds, nor do they correspond directly between (**A**) and (**B**).

A three-way separation was also revealed using correlation network analysis for both the community structure and functional potentials annotated with the COG database (Fig. 5). These clusters emerged when considering only strong, significant correlations (Spearman’s *r* > 0.5, *p* < 0.05), highlighting groupings of taxa and functions shaped by disturbance frequency.

## Discussion

Digesters that were disturbed at different frequencies at constant incidence separated in terms of community structure and functional traits. Ordination analysis (CAP, Fig. 1) revealed three distinct clusters aligned with the CSR life-history strategy triangle, indicating the emergence of competitor, ruderal and stress-tolerant strategies. They also resulted in trade-offs in process performance, as seen in the CAP biplot. Together, these results support the adoption of three-way life-history strategies by the communities under disturbance, similar to the CSR framework^12^. Ordination analyses have been previously employed to identify three-way life-history strategies in different environments, including activated sludge bacterial communities under disturbance^11,17^, fish communities^22^, coral reefs^23^ and soil microbiome^10^. To the best of our knowledge, the present study represents the first use of this framework in an open anaerobic digestion ecosystem using MAGs for assessing the community structure. The CSR classification encompassed 146 archaeal and bacterial genera, 25 ecosystem functions and 201 COG trait complexes.

We propose that different frequencies of disturbance, even at constant incidence, caused the adoption of different life-history strategies by the studied communities. The CSR strategies—competitor, ruderal and stress-tolerant—were adopted by undisturbed, intermediately disturbed and press disturbed communities, respectively. The community-level functions were distinct across categories: the highest biogas yield (the most efficient conversion of organics to biogas) was observed in competitors; soluble COD removal was highest in ruderals; and the VS content (equivalent to biomass) in reactors was higher in competitors and ruderals than in the communities categorised as stress-tolerant (highest frequency of disturbance). Although the disturbance applied did not induce system failure (Table 1) or sustained deterioration in reactor performance, it resulted in clear and reproducible shifts in community structure, functional profiles and ecological strategies. Responses reveal that the disturbance affected the microbial community without causing process instability. This highlights that in microbial ecosystems, disturbances do not necessarily manifest as process failure, but can all the same drive a restructuring of community organisation and functional allocation. The adoption of three-way life-history strategies was also evident from the abundance of genes annotated with COG or CAZy categories in communities disturbed at different frequencies of disturbance. These patterns were supported by COG and CAZy gene abundances (Figs. 4, S5, S6), network correlations (Fig. 5), and Welch-ANOVA comparisons (Tables S5, S6), highlighting functional trade-offs aligned with life-history strategies. The observed pattern in the correlation network (Fig. 5) represents emergent modular structure derived from unsupervised correlation analysis across taxa and functions and should not be interpreted as a direct partition into CSR categories or trophic guilds. While anaerobic digestion systems are organised around established trophic roles (e.g. hydrolysers, fermenters, methanogens), taxa within the same functional guild may exhibit different responses to disturbance and therefore occupy different positions within the CSR framework. For example, methanogenic taxa were not assigned uniformly to a single CSR category, indicating that shared metabolic roles do not imply shared ecological strategies under disturbance. The CSR classification thus provides an additional ecological dimension, capturing differences in disturbance-response strategies, functional trade-offs and stability that are not resolved by trophic grouping alone.

**Fig. 6:**
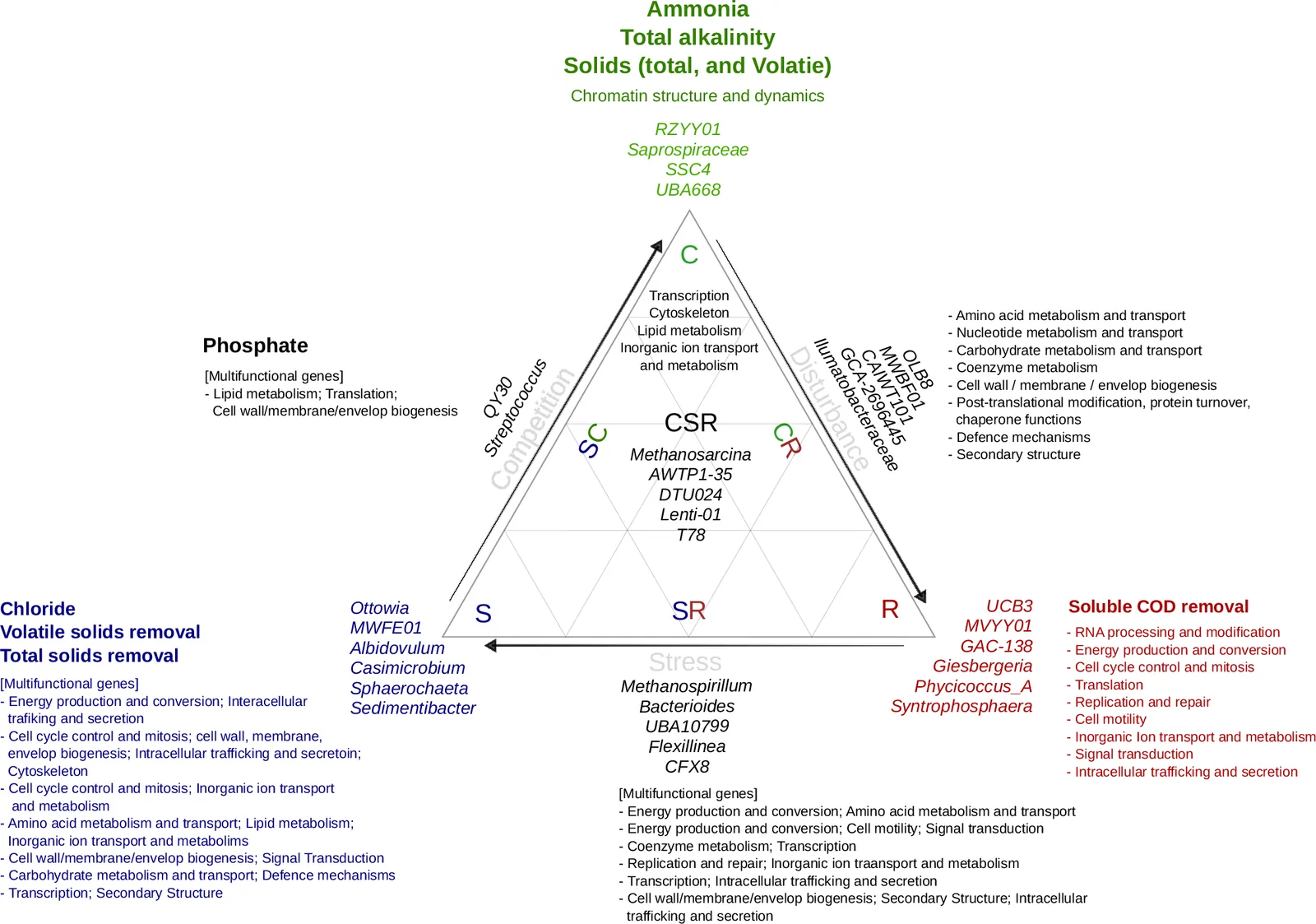
Distribution of taxa, COG functional categories and community-level functions in an anaerobic digestion ecosystem under varied frequencies of disturbance explained by the CSR framework. Traits classified under C, S and R categories are shown in green, blue and red colours, respectively. C competitor; S stress-tolerant; R ruderal. Multi-functional genes are genes assigned to more than one COG category.

Following the approach of Santillan et al.^11^, the community structure, potential functions and community-level traits were combined into a conceptual CSR framework (Fig. 6). While our study explicitly manipulated disturbance frequency, we recognise—consistent with Grime’s original formulation^12^—that CSR strategies arise from a combination of three ecological forces: disturbance, stress and competition^11^. Different disturbance regimes can indirectly reflect these forces noted before^11^. For example, undisturbed reactors may reflect high competition due to resource accumulation and low diversity, while press-disturbed reactors may represent high-stress environments due to chronic biomass loss. Our schematic thus integrates this broader interpretation, providing a holistic representation of the ecological strategies adopted under varying conditions.

Interpretation of functional traits derived from metagenome-assembled genomes requires acknowledging that genomic trait content reflects potential energetic investment and allocation strategies rather than realised phenotypes at the level of individual organisms. Within the CSR framework, life-history strategies emerge from trade-offs in how limited resources are allocated among growth, maintenance, stress tolerance and resource acquisition. Under sustained disturbance regimes, selection is therefore expected to favour the enrichment of specific functional gene categories that maximise fitness under those conditions. Accordingly, we interpret differential enrichment of COG categories as community-level indicators of dominant life-history strategies, rather than as organism-specific metabolic capabilities.

Community-level interpretation of functional traits has previously been explored in microbial ecology as a way to infer dominant life-history strategies when individual-level phenotypes cannot be directly measured. In activated sludge systems, trait-based frameworks have been applied using community-aggregated functional annotations to reveal disturbance-driven trade-offs among microbial strategies^11^^,17^. More recently, similar conceptual approaches have been used in soil microbial communities to interpret functional gene enrichment patterns along environmental gradients^10^.

Importantly, these earlier studies relied on marker-gene or metagenome-wide functional profiles and did not employ genome-resolved metagenomics to link functional traits to reconstructed microbial populations. In contrast, the present study represents the first application of a trait-based life-history framework using genome-resolved metagenomic data, enabling functional traits to be interpreted in the context of reconstructed genomes while retaining a community-level perspective. By applying this approach to anaerobic digestion systems—an ecosystem with distinct energetic constraints and disturbance dynamics—we extend both the methodological and ecological scope of trait-based microbial life-history analysis.

Beyond assigning organisms to ecological categories, the CSR framework provides a lens for interpreting how microbial communities organise under repeated disturbance. In the present study, disturbance frequency at a constant number of disturbance events did not result in a linear or gradual shift in community composition. Instead, communities clustered into distinct life-history strategies characterised by differences in dominance structure, functional trade-offs and variability in ecosystem performance. This framework allows observed differences in biogas yield, biomass retention and functional stability to be interpreted mechanistically in terms of alternative ecological strategies, rather than as isolated taxonomic or functional changes.

The high efficiency proposed by Grime and Pierce^24^ as one of the main indicators of competitive communities was observed in undisturbed communities in this study. Undisturbed reactors showed the highest biogas yield, suggesting better performance compared to reactors under disturbance conditions. In addition, the volatile solids (VS) content of the reactors (as a measure of biomass) was the highest in undisturbed reactors. This trait has also been proposed as one of the C-traits in activated sludge bacterial communities^11^ and in mycorrhizal fungal ecosystems^15^. The genotypic functional analysis showed that genes assigned to more than one COG category (multi-functional genes) were dominant in undisturbed communities. These patterns are interpreted as differences in functional allocation at the community level, rather than as evidence for the presence of unique or exclusive metabolic functions. These multi-functional genes are responsible for energy production and conversion, amino acid metabolism and transport, coenzyme metabolism, carbohydrate metabolism and transport, cell cycle and mitosis and cell wall/membrane/envelop biogenesis. Similar functional traits were reported before as C-traits^11^. Genes assigned to chromatin structure and dynamics were dominant in communities classified as competitor. This function has been reported to be related to growth rate^25^. Dominance of several taxa has been proposed as an indicator of competition^11^. Here, we observed that the undisturbed communities were dominated by several taxa—namely the genera *RZYY01, Saprospiraceae, SSC4 and UBA668*—whereas communities subjected to intermediate frequencies of disturbance were dominated by only one genus (*Syntrophosphaera*) with an average relative abundance of 14%.

Ruderals are known for inefficiency in energy uptake^24,26^, higher variability in ecosystem functions^11,26^ and increased dissimilarities of communities across replicates exposed to the same frequency of disturbance^11,26^. The reactors exposed to intermediate frequencies of disturbance in this study showed higher variability in terms of ecosystem functions (Table 1). In addition, the distances between replicate communities exposed to intermediate levels of disturbance in ordination analysis were greater than at the extreme ends of the disturbance range. Furthermore, it has been suggested that intermediate levels of disturbance create opportunities for less abundant community members, that is, seed-bank species, to outcompete dominant community members^11,27^. The emergence of the genera including *Methanosprillium*, *Accumulibacter*, *GCA-2748155*, *Rectinema* and *MWFE01* was observed in disturbed reactors, while they were not abundant in undisturbed communities. Another reported trait for the ruderal category is a high biomass growth rate^11^. The VS content in intermediately disturbed reactors was similar, despite having been disturbed at different frequencies. Considering the nature of the disturbance applied (biomass removal), biomass growth should have been promoted in reactors disturbed at a medium frequency for the VS content to be similar to the reactors disturbed at low disturbance frequency. This observation is consistent with Santillan et al.^11^ who reported higher biomass in the intermediately disturbed activated sludge communities compared to the undisturbed and press disturbed reactors. In a soil microbiome study^28^, high growth rate and yield were classified as copiotroph and oligotroph traits, respectively. Based on that study^28^, Krause et al.^14^ categorised high growth rate as a competitor trait, which contrasts with the observation in this study.

Analysis of genotypic functional traits revealed that genes annotated with a single COG category (specialised genes) were more abundant in communities exposed to intermediate disturbance frequencies (low and medium). This aligns with the notion that ruderals are less efficient^14,26^, requiring a broader set of genes to perform essential functions. In contrast, competitors may rely on multifunctional genes to perform similar functions. Accordingly, broad functional categories such as energy production and conversion, translation, replication and repair, signal transduction, intracellular trafficking and secretion, cell cycle and control and cell motility were relatively more abundant in the communities classified as ruderal. Importantly, these categories are not interpreted individually as mechanistic indicators of specific traits, but as components of aggregate, community-level functional profiles^1,2,10,17^. Their enrichment reflects relative shifts in functional allocation across disturbance regimes, arising from trade-offs within and among taxa consistent with CSR theory, rather than differences in the capacity to perform these essential processes. Cell motility represents a point of contention in studies applying trait-based frameworks. Consistent with our observation, Krause et al.^14^ categorised it as a ruderal trait, while Santillan et al.^11^ found it to be enriched in stress-tolerant activated sludge communities. It was suggested that these traits are shared between S and R categories^11,26^. These discrepancies highlight the context-dependent nature of trait assignments and the need for robust, reproducible classification approaches.

Microorganisms in high-stress environments are known to allocate more energy towards maintenance-related functions, such as repair and homoeostasis, which can reduce their capacity for growth and biomass production^11,14^. In this study, highly disturbed communities exhibited the lowest biomass among all. While this may reflect stress-induced physiological trade-offs, the direct removal of biomass as part of the disturbance complicates casual attribution.

Remarkably, the genes assigned to multiple COG categories (multi-functional genes) were more abundant in reactors exposed to a high frequency of disturbance. These included COGs related to cell cycle control and mitosis, intracellular trafficking and secretion, cell wall/membrane/envelop biogenesis and inorganic ion transporters. Such multifunctional traits (membrane chemistry and uptake system^14^, transport and ATP binding cassette transporter genes^11^) have been associated with stress-tolerant strategies.

We acknowledge that broad COG categories provide a coarse functional resolution and may be influenced by genome size and taxonomic composition. As such, enrichment of individual categories (e.g. cell cycle, translation) should not be interpreted in isolation as evidence of specific metabolic capabilities. Instead, these categories are used as coarse descriptors of community-level functional allocation patterns, in line with CAT-based approaches that capture the collective functional potential of microbial communities rather than individual taxa^2,11,17^. While genome size and taxonomic composition may contribute to observed patterns, our conclusions are based on consistent shifts across treatments, supported by replicate agreement and independent ecosystem-level measurements. Future work incorporating finer-scale functional annotation and explicit normalisation for genomic features may further refine these interpretations.

The majority of the microorganisms and genotypic traits in this study were classified into inter-categories, namely, CR, CS, RS, or CSR. Intermediate classifications (e.g. CR, RS) reflect functional overlap or shared adaptive strategies between life-history categories. Inter-category traits have been reported in previous studies, for example, classification of cellular component organisation^11^ under the CR category, chemotaxis^11^ and sporulation^11^ related functions as SR and hyphal fusion^15,29^, growth rate^15,30^ and cell division^11^ as CS. Additionally, some traits—such as cell motility^11,14^ and sporulation^11,14,15^—have been classified under different categories in different studies. These discrepancies may have different roots; those traits arise because such traits are selected under multiple ecological pressures (for example, high disturbance and high stress), or due to methodological issues such as arbitrary cutoffs or insufficient statistical power. To overcome the classification errors, we employed the CSR_Simulation function of the MicroEcoTools package^19^, which enables quantitative and reproducible assignment of traits to CSR categories through robust statistical comparisons across experimental groups.

Given the limited replication common in experimental studies, we also applied the CSR_Simulation function in MicroEcoTools, which uses abundance-derived probability distributions to simulate communities and estimate classification confidence. While most simulation results validated the original classifications, they also revealed cases of potential reclassification under minor abundance shifts. For example, *Albidovulum* (originally R) was classified as CSR in 20% of simulations, and a *Rhodanobacteraceae* genus (CSR) was classified as S in 20% of cases. Functional traits showed even greater variability; for example, RNA processing and modification was classified as R based on observed data, but simulations suggested only a 20% probability of it truly belonging to R. These results underscore the value of in silico methods for strengthening trait classification, particularly in datasets with limited experimental replicates^19^.

This study demonstrates that life-history strategy frameworks, particularly Grime’s CSR model, provide a powerful conceptual tool for interpreting microbial community responses to disturbance in engineered ecosystems. By applying the CSR framework to anaerobic digestion—an ecologically and industrially important yet understudied ecosystem—we show that disturbance frequency at constant incident number can drive the emergence of distinct community strategies that align with competitive, ruderal and stress-tolerant life-history patterns. Importantly, the CSR framework enables interpretation of these patterns in terms of functional trade-offs, dominance structure and variability in ecosystem performance, rather than simple taxonomic or functional inventories. In the present study, these strategies were associated with distinct performance patterns, where competitor communities link to higher biomass retention, ruderal communities to increased variability and stress-tolerant communities to reduced biomass under frequent disturbance. Trait-based approaches, through the lens of CSR theory, allow for a more mechanistic understanding of community organisation and functional trade-offs in response to disturbance. The identification of shared and intermediate strategies further reflects the complexity and adaptability of microbial life in dynamic environments. Because the CSR framework is independent of taxonomic identity, it provides a transferable, theory-driven approach for comparing microbial communities across engineered and natural systems that experience disturbance. Beyond ecological interpretation, these findings suggest that community-level trait profiles may offer a useful lens for understanding process stability, variability and resilience in anaerobic digestion systems. These observations further suggest that shifts in CSR positioning could provide early indications of changes in process behaviour, potentially preceding measurable declines in performance. In this context, CSR-based characterisation may support the development of microbiome-informed monitoring strategies to identify system imbalance and inform operational control. Accordingly, the CSR framework provides a foundation for linking microbial community structure to process performance, with potential applications in monitoring, prediction and control of engineered microbial systems. As the application of trait-based frameworks continues to expand in microbial systems, our study contributes to establishing their ecological validity in anaerobic systems and underscores their potential for guiding future research on microbial community resilience, succession and ecosystem function in engineered settings.

## Methods

### Experimental design

A set of twelve laboratory-scale continuously stirred tank reactor anaerobic digesters with a working volume of 4 L was operated under mesophilic conditions (35 °C). These digesters were inoculated with anaerobic sludge obtained from a full-scale mesophilic anaerobic digester treating a 1:1 mixture of primary and waste activated sludge (PS and WAS) at an SRT of 15–25 days. The inoculum had a total solids (TS) and VS content of 24.05 g/L and 17.08 g/L, respectively. After inoculation, the solids retention time (SRT) was reduced from 15–25 to 10 days.

To acclimate the sludge to the new conditions, digesters were operated for a period of 7 months prior to the experiment. After acclimation, sludge was extracted from all digesters, mixed and redistributed among digesters with minimum air exposure. After that, a second month-long acclimation was performed to ensure the operational stability of digesters. The average total chemical oxygen demand of the feed sludge for the lab-scale digesters was 95.67 g/L.

The acclimated digesters were then assigned to four experimental groups, each undergoing five disturbance events at varied frequencies (Fig. S1). Disturbance was operationally defined as controlled biomass removal events. Under baseline (undisturbed) conditions, reactors were operated at a constant SRT by daily replacement of 400 mL of anaerobic sludge (mixed liquor) with an equal volume of influent feed. During disturbance events, 800 mL of anaerobic sludge (twice the daily exchange volume) was removed and replaced with 400 mL of influent feed and 400 mL of deionized water to maintain a constant reactor volume. This resulted in a transient reduction in biomass concentration without altering the overall volumetric balance of the system or the organic loading rate.

Disturbance frequency was varied across treatments while maintaining a constant total number of disturbance events. We note that biomass removal inherently affects multiple system properties, including the biomass concentration and food-to-microorganism (F/M) ratio; in this study, disturbance is defined operationally as biomass removal and interpreted in terms of its combined ecological effects.

Throughout the experiment, daily sampling was done to assess the performance of the digesters. Samples were analysed on the same day for COD (soluble and total), solids (total and volatile), cations and anions and biogas composition. To assess the community structure and perform functional analysis of the metagenomes, one sample per digester was collected 10 days after the last disturbance event. For community analysis, samples were removed from each digester using syringes to avoid air exposure and transferred into cryo-vials containing ZymoResearch shield buffer (ZymoResearch, CA, USA) to instantly inactivate the microorganisms and prevent degradation of the nucleic acids. One sample per digester was collected for DNA extraction. These samples were stored at -80 °C until nucleic acid extraction.

### Physicochemical analysis

Ammonia and chemical oxygen demand analyses were performed using Hach kits (Hach COD HR and Hach TNT-plus ammonia HR kits [Hach, Loveland, CL, USA]) along with a Hach DR6000 spectrophotometer (Hach, Loveland, CL, USA). Solids (total, volatile and fixed) analysis was performed according to APHA AWA standard methods^31^. Volumetric biogas production rate was measured using Bioprocess Instruments uFlow flowmeters equipped with 9 mL flow-cells (BP Instruments, Lund, Sweden). Analyses of biogas and volatile fatty acids composition were performed on a Shimadzu GC-2010 plus (Kyoto, Japan). A Shimadzu LC-20AD (Shimadzu, Kyoto, Japan) instrument was utilised for determination of anion and cation concentrations.

### Nucleic acid extraction and metagenomics shotgun sequencing

For nucleic acid extraction, 0.5 mL of sludge was thawed and transferred into ZymoResearch bashing beads tubes (ZymoResearch, CA, USA) and subjected to 4 × 40 s at a speed of 6 m/s of beating using a FastPrep-24 5G MP Biomedicals bead beating machine (MP Biomedicals, Singapore). Samples were cooled on ice for 5 min after every beating cycle. After bead beating, samples were centrifuged at 15,000 × *g* for a duration of 10 min. Then, 0.2 mL of the supernatant was used to purify DNA using the ZymoResearch MagBead DNA/RNA extraction kit (ZymoResearch, CA, USA). An additional ethanol wash step was added to the manufacturer’s manual for improved contaminant removal. The nucleic acids were purified using an automated pipeline on a Tecan M200 Evo (Tecan, Männedorf, Switzerland) liquid handler. The quality of the extracted DNA was assessed using a Nanodrop (Thermo Fisher Scientific, MA, USA), a Qubit 4 (Thermo Fisher Scientific, MA, USA) with the broad range double-stranded DNA kit and an Agilent Tapestation (Agilent technologies, CA, USA) using the genomic DNA tape. Library preparation was performed using the Illumina TruSeq nano library preparation kit (Illumina, CA, USA). The libraries were sequenced on an Illumina Hiseq-X sequencing platform (Illumina, CA, USA) to obtain about 30 M 150 bp paired-end reads per sample.

### Genome-resolved bioinformatics and functional analysis

Changes in the microbial communities were assessed using a genome-resolved bioinformatics pipeline^32^. Briefly, raw reads were trimmed to remove sequencing adaptors and low-quality reads using Trimmomatic v0.36^33^ in paired-end mode (default parameters except for PE LEADING:3 TRAILING:3 SLIDINGWINDOW:4:15 MINLEN:36). Given the high base quality of Illumina HiSeq-X data, FastQC confirmed that the vast majority of reads exceeded Q30 across their length; trimming therefore served primarily to remove adaptors and residual low-quality bases rather than to enforce stringent quality filtering. Quality control for reads before and after trimming was performed using FastQC^34^. Trimmed reads from replicates in the same experimental group were assembled into contigs using Spades v3.13 in metagenomic mode using—meta flag^35^. After that, the raw reads were mapped onto the assembled contigs using BBMap^36^. The mapping results were summarised using a Metabat2 function^37^ (jgi_summarize_bam_contig_depths function). Metabat2^37^ was used to generate genome bins using coverage stats with default parameters. While combining outputs from multiple binning algorithms can increase genome recovery in catalogue-focused studies, such approaches also require consensus strategies and post-hoc curation that introduce additional assumptions and potential subjectivity. Because the primary objective of this study is comparative, trait-based analysis of community responses across disturbance regimes, rather than exhaustive genome reconstruction, we intentionally employed a single, well-established binning algorithm^38,39^ across all samples. This approach minimises methodological complexity and ensures that observed ecological patterns are not confounded by binning-model heterogeneity or consensus selection effects. Generated metagenome-assembled genomes (MAGs) were dereplicated and quality filtered using dRep v2.3.2^40^ (lineage workflow with -nc 0.5 -pa 0.9 -sa 0.99 -comp 50 -con 10). The completeness and contamination filtering performed in dRep corresponds to the MIMAG^41^ medium quality criteria (completeness > 50% and contamination < 10%). Taxonomy classification was performed using GTDB-tk v1.5 with GTDB release 207^42^. Relative abundances based on coverage were calculated using CoverM^43^ on genome mode using 20 M subsampled reads with mappings performed using BBMap software package^36^. Functional annotation was conducted using eggNOG-mapper v2.1.2^44^ and the eggNOG 5.0^45^ database on the multi-FASTA files (Bowers et al.^41^). Seal function from BBMap^36^ package was utilised to calculate the coverage of the annotated open reading frames (ORF) predicted by prodigal in eggNOG-mapper run (using 20 M subsampled reads). Annotated ORFs from all retained MAGs were combined into a unified reference to generate coverage-weighted, community-level functional profiles, enabling comparison of MAG-derived functional traits across disturbance regimes. Briefly, annotated ORFs and mapping results were joined by ORF tags. After that, coverage was used as the weight variable to calculate the weighted counts for the cluster of orthologs genes (COG^46^) and CAZy^47^ categories, previously described as CATs^11^.

### Statistical analysis

R v4.0.0^48^ running on an Apple Mac OS Darwin 17.0 was utilised to perform all statistical analyses.

### Competitor–stress-tolerant–ruderal category assignment

MicroEcoTools^19^, a computational tool for applying microbial ecology frameworks on computational data, was utilised to classify MAGs and functional categories under the CSR framework. The CSR_assign function of the MicroEcoTools assigns competitor, stress-tolerant and ruderal categories to organisms and functional traits abundant in the absence of, under intermediate and high levels of disturbance, respectively. Briefly, for each MAG or functional trait, abundance profiles across disturbance treatments were first tested for significant enrichment using Welch’s ANOVA (*p* < 0.05). Traits or organisms that were not significantly enriched in any experimental group were classified as CSR. If significant enrichment was detected, classification proceeded based on the disturbance regime(s) in which enrichment occurred.

Entities significantly enriched in only one group were classified as competitors (C) if enriched in undisturbed reactors, ruderals (R) if enriched under intermediate disturbance, or stress-tolerant (S) if enriched under high disturbance. When significant enrichment occurred in two disturbance regimes, inter-category assignments were made: competitor–ruderal (CR) for enrichment in undisturbed and intermediate disturbance, competitor–stress-tolerant (CS) for enrichment in undisturbed and highly disturbed reactors, and stress-tolerant–ruderal (SR) for enrichment in intermediate and highly disturbed reactors.

### Statistical comparison tests

For uni-variate comparison, Welch-ANOVA^49^ was performed with *p*-value adjustment using the Benjamini-Hochberg method^50^, implemented via the MicroEcoTools R package. In cases where the variance in at least one group was zero, ANOVA was employed to test the significance.

### Correlation analysis

Correlation analysis (Spearman’s rank) on community (coverage), functional (coverage weighted counts) and physicochemical data was performed using the Hmisc v4.6.0^51^ R package. *P*-value correction for multiple comparisons was performed using the Benjamini-Hochberg method. Results of the correlation analysis were plotted for pairs with significant correlations (adjusted *p* < 0.05). Cluster analysis (hierarchical clustering) was performed using the hclust base R function. For correlation analyses, MAG abundances were aggregated at the highest confidently resolved taxonomic rank (typically genus, or family where genus-level assignment was not supported) to reduce strain-level redundancy and improve ecological interpretability.

### Heatmaps

For heatmaps showing MAG abundances, tile depths represent the relative abundance of each MAG (y-axis) in each digester (x-axis). For COG and CAZy heatmaps, the weighted count of each category in each digester is shown as tile depth. In these plots, the x-axis indicates disturbance frequency and the y-axis lists functional categories. To highlight the differences in each functional category (in COG and CAZy heatmaps), data were scaled per category. For all heatmaps, hierarchical double clustering was applied. Heatmaps were plotted using the pretty heatmaps (pheatmap)^52^ R package.

### Multivariate analysis

Permutational multivariate analysis of variances (PERMANOVA) was employed to compare the community and functional compositions of the digesters across experimental groups. For this analysis, the adonis function of vegan v2.5.7^53^ R package using Bray-Curtis dissimilarity matrix was used with 999 permutations. The betadisper function in the same R package was used to assess the homogeneity of dispersion^21^. Constrained canonical analysis of principal coordinates (CAP) was performed to compare the communities under different disturbance levels. Physicochemical data were fitted to the CAP results and plotted as a biplot (only significant correlations are plotted).

### Network analysis

To assess the relationships between the community members and functional categories, correlation network analysis was performed. Network graph construction and visualisation was done using igraph v1.2.11^54^ and visNetwork v2.1.0^55^ R packages. Generated network graphs were exported into high-quality vector format using visNetwork-to-SVG online tool (https://soheil-a-neshat.github.io/visNetwork-to-SVG).

## Supplementary information

Supplementary files

## Data Availability

DNA sequencing data are available in the NCBI BioProject database under accession number PRJNA723443. Genome-resolved metagenomic outputs, including MAG taxonomy, coverage-based relative abundance and COG count data supporting the analyses in this study, are available via Zenodo at https://doi.org/10.5281/zenodo.19550057^20^.
