# Supplementary material for: Uncovering microbial life-history strategies under disturbance: a trait-based computational analysis of anaerobic systems
Source: NPJ Biofilms Microbiomes. 2026 Jul 10;12:220. doi: 10.1038/s41522-026-01067-8 (PMC13728152; doi:10.1038/s41522-026-01067-8)
Supplement: Supplementary file 1 [file 41522_2026_1067_MOESM1_ESM.zip › 20260414_Neshat_etal_Fig2_interactive.html]

Fig. 2 – CSR Life History Strategies in Anaerobic Digestion Communities


# Figure 2 — Life History Strategy (CSR) of Dominant Taxa Across Disturbance Frequencies in Anaerobic Digesters

Hover or click each panel to reveal CSR assignment, LOESS trend, and statistics  ·  Relative abundance (%) on Y-axis  ·  Disturbance frequency on X-axis

Relative abundance (%)

◈ Show All Trends

Taxa shown Fig 6
25 taxa (top abundant)
50 taxa
100 taxa
All (146 taxa)

● Scatter plot
▬ Bars + SD

⬇ Export ▾

SVG (vector)
JPEG (raster)
TIFF (raster)

Disturbance frequency

#### Disturbance frequency (colour)

0 — Control

0.2 — Low

0.4 — Intermediate

1.0 — High

#### Reactor (shape)

C1

C2

C3

H1

H2

H3

I1

I2

I3

L1

L2

L3

#### CSR strategies

BH-adjusted p-values from Kruskal-Wallis test across disturbance groups  ·  \* indicates statistical significance (p < 0.05)  ·  Trend line = cubic spline through group means
